# An Act to Establish a State Board of Medical Examiners and Licensers

**Published:** 1885-08

**Authors:** 


### AN ACT TO ESTABLISH A STATE BOARD OF MEDICAL EXAMINERS AND LICENSERS AND TO DEFINE THE DUTIES AND POWER OF SUCH BOARD.

---

At the last meeting of the American Medical Association, the following bill was prepared and ordered sent to the various State Associations, requesting that they report on the bill at the next meeting of the American Medical Association. We give the bill entire, that the Medical Association of Georgia may be prepared by the next meeting of that body to act on it:

Section I. Be it enacted by the Senate and House of Representatives of the Commonwealth of ——————— in General Assembly met, and it is hereby enacted by the authority of the same, That there shall be appointed by the Governor a State Board of Medical Examiners and Licensers, consisting of nine members, three of whom shall serve for one year, three for two years and three for three years, and hereafter he shall each year appoint three members to serve for three years, in place of those whose terms then expire. They shall be graduates of some legally chartered college or university, having the power to confer medical degrees, who shall have practiced medicine or surgery for a period of not less than five years, but none of whom shall be members of the faculty of any such college or university. Provided, that in the appointment of said Board, at least seven members shall be chosen from a list of twenty-one names, submitted by the Medical Society of the State of——————— In default of the submission of such list the Governor shall appoint ———————

Sec. II. Upon the organization of the said Board, it shall be determined by lot which three members shall serve for a term of one year, which three for a term of two years, and which three for a term of three years. Every appointment to fill a vacancy or vacancies in the said State Board of Medical Examiners and Licensers shall be for the unexpired term, and the said vacancy or vacancies shall be filled by the Governor within sixty days after notice to him of the same. Provided, that when the vacancy has been caused by death, resignation or removal of a member appointed from the list furnished by the Medical Society of the State of ————————, the said vacancy shall again be filled from a list of three names for each vacancy furnished by the Medical Society of the State of ———————— In default of the submission of such list the Governor shall appoint————————

SEC. III. The said Board shall be a corporation by the name and style of The State Board of Medical Examiners and Licensers of the Commonwealth of ————————, and shall have and use a common seal, and as such corporation may sue and be sued, contract and be contracted with, plead and be impleaded to the extent to enable it to carry out the powers conferred upon it by this Act. Said Board may make and adopt all necessary rules, regulations and by-laws, not inconsistent with the Constitution and laws of this Commonwealth, or of the United States, to enable it to perform its duties and transact its business under the provisions of this Act, and shall upon its organization elect from its own number a President and a Secretary, who shall also act as Treasurer, both of whom shall hold their offices for a term of two years.

SEC. IV. Every person who shall be appointed to serve on the said State Board of Medical Examiners and Licensers, in the manner aforesaid, shall receive a certificate of appointment from the Governor, and within thirty days after receiving the certificate of appointment shall file the same with the Prothonotary of the Court of Common Pleas in the county in which he or she shall have previously registered under the present Acts of Assembly, and shall also file a certificate of his or her said appointment as a member of said State Board of Medical Examiners and Licensers in the office of the Secretary of State of the Commonwealth of ——————

Sec. V. The said State Board of Examiners and Licensers shall examine all applicants for license to practice medicine or surgery in this Commonwealth, who are properly qualified, according to the provisions of Section VII. of this Act, and shall exclude no one from examination, nor reject him or her because of his or her adhesion to a special system of practice. It shall hold two stated meetings each year, one at ———, on the second Tuesday in May, and one at ———, on the second Tuesday in November respectively, and may hold special meetings at such times as it may deem proper. All examinations shall be conducted in writing, and all examination papers, together with the reports and action of the Examiners thereon, shall be preserved as the records of the said Board for a period of five years, during which time they shall remain open for inspection at the office of the said State Board of Medical Examiners and Licensers, which office shall be in ——————

Sec. VI. Such examinations shall be in anatomy, physiology, general chemistry, pathology, therapeutics, principles and practice of medicine, surgery and obstetrics. Provided, that each applicant, upon receiving from the Secretary of the Board an order for examination, shall receive also a confidential number, which he or she shall place upon his or her examination papers, so that when said papers are passed upon by the Examiner, the latter shall not know by what applicant said papers have been prepared; that upon each day ot examination all candidates be given the same set or sets of questions. It is further provided that the examination papers shall be marked upon a scale of one hundred, and that, in order to secure a license, it shall be necessary for the applicant to attain such average as shall hereafter be determined by the said State Board of Examiners and Licensers.

SEC. VII. Any person, on paying twenty dollars to the Secretary of the said Board, and on presenting satisfactory proof of being over twenty-one years of age, of good moral character, and of having received a diploma from any legally chartered medical college or university having authority to confer degrees in medicine, shall be entitled to examination by the said Board, and, in case of failure at any such examination, shall have the privilege of a second examination without the payment of any additional fee.

SEC. VIII. For the purpose of examining and licensing applicants, as well as for the transaction of other business, five members shall constitute a quorum of said Board, and when the President and Secretary of said Board shall find that an applicant has attained the necessary examination average, they shall issue to him or her a license to practice medicine and surgery in the State of

——————

SEC. IX.—After the first day of September, 1886, no person shall enter upon the practice of medicine or surgery in the State of ——————, unless he or she has complied with the provisions of this Act, and shall have exhibited to the prothonotary of the Court of Common Pleas of the county in which he or she resides, a license duly granted to him or her by the said State Board of Examiners and Licensers, upon which he or she shall be entitled, upon the payment of one dollar, to be duly registered in the office of the prothonotary of the Court of Common Pleas in said county, but nothing in this Act shall be so construed as to prevent the practice of medicine and surgery by any practitioner who shall have been duly registered before the first day of September, 1886, according to the terms of the present Act of Assembly.

SEC. X. For the purpose of this Act the words "practice medicine or surgery" shall mean to treat or attend any person for money, gift or reward.

SEC. XI. Nothing in this Act shall apply to commissioned medical officers of the United States army or navy, or of the United States Marine Hospital service, nor to any member of the house or resident staff of any legally chartered medical college or university or hospital, during his term of service therein, nor to physicians of other States meeting duly registered physicians of this State in consultation, nor to those practicing dentistry exclusively, nor to midwives.

SEC. XII. The Secretary shall record, in a book to be kept for the purpose in the office of the said Board, the name, age, sex, residence, date and place of graduation of each applicant, together with the date of the examination, the examination number, the examination average on each branch, the general average and date of issue of license, in case such license is granted. Said book shall be open to public inspection, and on or before the last day of December of each and every year, the said Board shall publish, or cause to be published, a list of the names and addresses of such persons as shall have received licenses from the said Board within twelve months immediately thereto preceding.

SEC. XIII. The members of the said Board shall each receive a salary, not exceeding seven hundred dollars per annum, to be paid out of the fees for examination. The Secretary and Treasurer shall receive an additional salary, to be fixed by the Board, and shall give bond, in the sum of one thousand dollars, that he or she will faithfully account for the sums paid into his or her hands. The balance of the fees, after the necessary expenses of the Board, which must be stated by affidavit, have been deducted, shall be paid into the treasury of the Commonwealth of ———————

SEC. XIV. The Governor may remove any member of the said Board for unprofessional or dishonorable conduct upon the recommendation of a two-thirds vote of the said Board.

SEC. XV. The sum of one thousand dollars is hereby appropriated to meet the necessary and legitimate expenses of the said Board, for the year commencing the first day of September, 1886.

SEC. XVI. This Act shall take effect on the first day of September one thousand eight hundred and eighty-six.

SEC. XVII. Should any charge or charges of unprofessional conduct be preferred against any duly licensed practitioner of medicine or surgery, the said State Board of Medical Examiners and Licensers of the said Commonwealth of ———— shall have power to summon said practitioner before it, having previously given him or her fifteen days' notice of such charge or charges, with the name or names of the party or parties preferring the same, and the name or names of any witness or witnesses to be called and examined in support of said charge or charges, and upon hearing thereof, and, having heard the party or parties so accused, and any witness or witnesses he or she may desire to call as to his or her defence, the said State Board of Medical Examiners and Licensers shall, upon satisfactory proof of the truth of said charge or charges, refer his or her case to the District Attorney of the county wherein he or she shall have practiced, with all specifications and all evidence in support of the same, who shall apply for a rule upon the party so accused, to show cause why his or her license shall not be revoked, and the proper court shall have power so to direct such licensee to be stricken from the list recorded in the office of the Prothonotary of the Court of Common Pleas for the county in which the said accused shall reside.

SEC. XVIII. Any person violating the provisions of this Act shall be guilty of a midemeanor, and upon conviction thereof in the Court of Quarter Sessions of the county where the offense shall have been committed, shall pay a fine of not less than fifty nor more than five hundred dollars for each offense.

